# Effects of Mixed-Gender Competition: Choking under Pressure in a Dynamic Tournament

**DOI:** 10.3390/ijerph19084925

**Published:** 2022-04-18

**Authors:** Jungwon Min

**Affiliations:** College of Business Administration, Inha University, 100 Inha-ro, Michuhol-gu, Incheon 22212, Korea; jmin@inha.ac.kr

**Keywords:** mixed-gender competition, dynamic tournament, choking under pressure, gender equality, mistakes, task executions, figure skating competition

## Abstract

In sports, there has been a recent and active movement to promote mixed-gender competitions for achieving gender equality in the field. However, the current debate regarding its effects limitedly focuses on balancing the number of opportunities for females and neglects its effect on athlete performance. To address this gap, this study investigated whether and how mixed-gender competitions mitigate gender-specific disadvantages of interim leaders in dynamic tournaments from the perspective of choking under pressure. Using data from 127 international segregated-gender single and mixed-gender pair figure skating competitions organized by the International Staking Union from 1 July 2006 to 30 June 2018, this study showed that female interim leaders in segregated-gender competitions are more likely to make mistakes in task executions under pressure-inducing circumstances than male interim leaders. However, in mixed-gender competitions, all of these gender-specific influences disappear. The findings contribute to the literature on mixed-gender competitions by providing new evidence on the positive impact of them, as well as expanding the literature on the impact of gender on competitive pressure.

## 1. Introduction

To address gender inequality in sports, mixed-gender competitions have been a remarkable recent transformation. In such competitions, males and females compete together and against each other as a pair or team, thereby facilitating the equal participation of both genders. Several sports such as relays [1], golf [2] and volleyball [3] illustrate the shift, and have already attempted to incorporate mixed-gender competitions in their field. To accelerate this trend, the International Olympic Committee (IOC) has also adopted more mixed-gender competitions in the recent Olympic games [4].

However, despite this radical shift, an important question remains unanswered: Are mixed-gender competitions indeed effective in improving athletic competitiveness? Although further implementation of such competitions is required to speed up the application of this new approach, so far, they seem to place the most emphasis on merely increasing the number of female participants. As a result, there has been little discussion on whether and how mixed-gender competitions are effective in increasing the competitiveness of athletes, and consequently, how they contribute to achieving sustainable development in sport fields [5].

This study aimed to address this limitation by examining how mixed-gender competitions contribute to mitigating a gender-specific disadvantages in sports. As the gender-specific disadvantage, this study focused on a tendency of heightened competitive pressure on female sportspersons, which has received attention from scholars. Studies in diverse research areas such as economics, psychology or sociology, have found that gender inequality in competitions is rooted in gender differences under competitive pressure. According to these studies, regardless of their abilities, not only disfavor participation in pressure-inducing competitive events [6] but also underperform in such situations [7]. This tendency potentially harms sustainability in sports as it discounts the abilities of female athletes as well as the opportunities available to them. This study examined male and female professional athletes in segregated- and mixed-gender sports competitions to find out how they perform differently under pressure-inducing circumstances.

## 2. Theoretical Background

### 2.1. Choking under Pressure of Interim Leaders in Dynamic Tournaments

Individuals in a competition are expected to perform according to their qualifications and efforts. However, their actual performance may not necessarily reflect these. This is because individuals tend to underperform under competitive pressure due to “choking under pressure” [8], a concept that has long been discussed in interdisciplinary research. According to existing literature, choking under pressure occurs because of excessive [9] or insufficient attention [10] toward task executions and is generated by competitive pressure. This abnormal attention toward tasks induces negative feelings in individuals such as anxiety, fear and worry, and upsets their task execution processes, leading to choking under the pressure [11].

A dynamic tournament is a context that can induce choking under pressure. This is a type of competition where players receive the results of their interim performance before their rankings are finalized [12,13,14]. For example, in some athletic competitions such as figure skating, tennis and weightlifting, players are temporarily ranked on the basis of their interim performance and they receive final rankings on their duly set standard at the end of the competition. This rule of sharing interim results imposes great competitive pressure on players in their subsequent performance, particularly on the interim leaders who achieve high interim rankings, due to the following two reasons.

First, since the final rewards of dynamic tournaments are presented only to the top-rankers, after the interim results are declared, audiences tend to concentrate only on the performance of the interim leaders. This results in increased pressure on the players to satisfy audience expectations and perform under concentrated attention. Such expectations can generate abnormal attention toward tasks, leading to players choking under pressure.

Second, the high threat of status loss for interim leaders may also generate competitive pressure and cause choking. Several previous studies have identified loss aversion as a major source of choking under pressure [15]. For instance, Pettit, Yong and Spataro found that individuals facing the threat of status loss tend to generate more pressure while solving mathematical problems than those facing opportunities for status gains [16]. In sports competitions, players in weight-lifting competitions tend to choke when ranked closer to the top in tournaments [17] and professional golfers are more likely to miss shots in high-stakes competitions [18]. Based on these studies, in a given dynamic tournament, interim leaders are more sensitive to potential ranking drops than other players. This is because the losses they will face due to a ranking drop are greater than those for other players. This will cause them greater anxiety toward retaining their interim ranking. Given these considerations, it is reasonable to focus on the interim leaders in dynamic tournaments to test the effects of choking under pressure.

### 2.2. Gender Differences in Choking under Pressure

A major concern of literature on gender differences in competitions is to explain the underrepresentation of females in competitive positions in society. One stream of research argues that participation depends on the differing sensitivities of the genders toward competitive pressure. According to research in this stream, females not only disfavor participation in competitive environments such as tournaments [6,19] but also perform poorly in these environments [7,13,19]. This phenomenon has been explained in two ways. First, females innately have a greater tendency to avoid risk than males regardless of their ability. In several laboratory and field experiments as well as surveys, scholars have found that females favor piece rates over a tournament in the payment schemes used to compete [20,21]. The latter is a much more competitive environment because it is a winner-takes-all approach, whereby participants gain nothing if they fail to win. Second, males and females differ in their levels of confidence which is a key factor in achieving high performance in competitions [22,23]. According to Niederle and Vesterlund [19], approximately 30% more females than males do not consider themselves the best performers in their respective competitions.

Supporting these explanations, scholars have empirically found that females tend to underperform in situations of competitive pressure compared to males or that they perform well under pressure only in limited contexts. For instance, competitive incentive systems increase performance for males but not for females [24]. In a similar vein, Shurchkov showed that females perform better than males in low-pressure exams but underperform in high-pressure exams [25]. Cahlíková et al. also found that in their laboratory experiment based on Trier Social Stress Test, females subjected to heightened stress perform worse than males with the same level of stress [26]. Other research has found that females perform well in limited tasks, but only those that highlight their relative advantages over males [27].

Overall, interim leaders in a dynamic tournament are vulnerable to choking under pressure. Since females have greater sensitivity to competitive pressures than males, female interim leaders in dynamic tournaments are more likely to be subject to pressure than males when they compete under pressure-inducing circumstances.

## 3. Hypotheses

This study argues that the effects of choking under pressure by interim leaders in dynamic tournaments is dependent on the gender of players, with more pressure felt by female than male interim leaders. To examine the influence of gender difference in competitive pressure, this study investigated two pressure-inducing circumstances that induce choking under pressure, namely, home-field competition and the threat of ranking-drop.

Since increased audience attention on interim leaders can cause competitive pressure and result in choking under pressure, its effects tend to be the highest in competitions held at the interim leaders’ home field [28,29]. Several studies have found that players tend to perform worse in home-field competitions in sports. For instance, some scholars found that in the context of golf, scores of home players tend to deteriorate more than those of foreign players [30]. Others found that home teams are at a disadvantage in crucial games of hockey championships [31]. Similar tendencies were also found in Davis Cup tennis matches [32]. These studies argue that home disadvantage arises from the high expectations of supportive audiences that place players under great pressure to win.

Another pressure-inducing factor for interim leaders is the threat of ranking drops (i.e., status loss) by competitive trailing players. Using data from NASCAR’s Winston Cup Series, Bothner et al. found that drivers’ race crashes rates were higher when taking risks as a result of facing competitors who had a chance to surpass their final ranks in tournaments [13]. Such ranking-drop is most affected by the perceived ability of the player trailing the interim leaders most closely. Since the closest trailing player has the highest possibility of dropping the interim leader’s rank, they will be pressured when they perceive the trailing player’s high competitiveness. For instance, if the trailing player acquired the gold medal in a recent competition, the interim leaders might feel inferior compared to a top player and be choked in their subsequent task executions.

If female interim leaders are more sensitive to competitive pressure than males, the influences from these pressure-inducing circumstances will be more significant for female than male interim leaders in segregated-gender dynamic tournaments. That is, female interim leaders will make mistakes more than males under amplified audience attention at home-field competitions and in situations where a strong competitor trails them closely.

Thus, this study hypothesized that:

**Hypothesis 1 (H1):**  *In segregated-gender dynamic tournaments, female interim leaders are more likely to make task execution mistakes than male interim leaders in home-field competitions*.

**Hypothesis 2 (H2):**  *In segregated-gender dynamic tournaments, female interim leaders are more likely to make task execution mistakes than male interim leaders when trailed by a highly competitive player*.

This study expects that these disadvantages of female interim leaders under competitive pressure will be reduced in mixed-gender pair competitions. Given the assumption that female disadvantage from competitive pressure arises due to gender-specific reasons, its effects should be less significant in mixed-gender pair competitions where female-specific characteristics are less notable, given the presence of a male in the pair. Indeed, some previous studies have found that females perform well in environments that remove gender-originating inferiorities [33,34].

Furthermore, in mixed-gender pair competitions, players communicate more with each other, not only during the competition but also during practice sessions. The intimate social interactions needed to accomplish common tasks contribute to mitigating negative emotions such as tension or anxiety that might induce competitive pressure [35]. Scholars have found that such communications in the mixed-gender teams are more effective and productive than segregated-gender teams in the execution of tasks [36,37], though their impact on motor tasks have not been investigated.

Accordingly, this study hypothesizes:

**Hypothesis 3 (H3):**  *The mistakes in task execution by interim leaders in home-field competitions will be less significant in mixed- than segregated-gender dynamic tournaments*.

**Hypothesis 4 (H4):**  *The mistakes in task execution by interim leaders being trailed by highly competitive players will be less significant in mixed- than segregated-gender dynamic tournaments*.

## 4. Methods

### 4.1. Overview of Empirical Context and Definitions

To test these hypotheses, this study used data from international segregated-gender single and mixed-gender pair figure skating competitions organized by the International Skating Union (ISU). All regulations and rules pertaining to figure skating competitions are set by this organization, so the competitions organized by the ISU decide the most qualified figure skaters in the world. In this regard, the acquisition of medals in these competitions is a great incentive for skaters, which puts them under high pressure. The competitions include the Winter Olympic Games, the World Championships, the European Championships, the Four Continents Championships and nine kinds of Grand Prix Series Competitions. Apart from the Winter Olympic Games that are held every four years, the other competitions are annual events.

The sample period started from 1 July 2006 and ended on 30 June 2018. It comprised 127 single and pair competitions across 12 seasons. Season refers to the time period in which competitions are organized. It begins on July 1 and lasts until June 30 of the following year. Throughout a season, skaters repeatedly perform the same skating program. The rules and regulations are revised by the ISU each season. The sample did not include competitions held before 2006 because, in response to a 2002 judging controversy, the ISU formally adopted the new international judging system and made it mandatory for all competitions starting from the 2006 season.

Each competition consists of two subprograms, the short and free programs. Throughout the programs, skaters must execute four or five generic types of techniques in both the single and pair competitions. These include spins, step sequences, spirals, jumps and lifts. This study defines each of these techniques as a task in this context. To examine the task performance of skaters, this study focused on the element scores of each task as they directly and objectively measure how well skaters perform the tasks in the specified ways. The judging of the element score for each task first takes place on the base value, which indicates the execution standard (i.e., difficulty) established by the ISU and then on the quality of execution. This provides the Grade of Execution (GOE). The GOE score represents the degree to which the task execution is appropriate relative to the standard and is an integer in the range of −3 to +3, with the final GOE scores weighted by the task difficulty. Different tasks have varying difficulties, so each task has a different maximum or minimum GOE value. However, a negative GOE value always indicates an unmet standard, that is, execution mistakes, whereas positive values indicate excellent task execution. In addition, a zero indicates a neutral performance that is neither categorized as poor nor excellent. Therefore, irrespective of the task varieties, the signs of the GOE scores inform the skaters if they made mistakes in the task executions.

The Appendix A describes additional explanations about short/free programs and tasks, along with the suitability of the context to test an argument of this study.

### 4.2. Data Setting

For the analysis, this study developed three separate samples of male and female skaters in segregated-gender competitions and pair skaters in the mixed-gender competitions. It identified the skaters ranked in the three top-most positions in short programs in each of the competitions and defined them as interim leaders. Subsequently, the task performance of these interim leaders in the free program were observed. This limited focus is reasonable for defining interim leaders as the rewards are awarded only to the top-three skaters in the final rankings: gold for first, silver for second and bronze for third. The other skaters will feel less pressured than these top-three skaters because they have less to lose after the interim results. The limited focus is also useful for controlling for the possibility of poor performance due to lack of skills.

The unit of analysis in this study were the interim leaders’ tasks in the free program in each competition. Based on a valid premise that skaters avoid choosing tasks that they cannot execute well, the task performance captures the perceived threat more accurately than the program performance. The final dataset for the task performance of the interim leaders in free programs included 10,287 observations of 52 male, 51 female and 48 pair skaters across 127 competitions. The tasks executed by single competition skaters included 117 kinds of jumps, 52 kinds of spins, 11 kinds of spirals and 8 kinds of step sequences. Those executed by pair competition skaters include 170 kinds of jumps, 62 kinds of spins, 22 kinds of spirals, 12 kinds of step sequences and 76 kinds of lifts. All data were taken from the ISU website (http://www.isu.org/figure-skating, accessed on 9 March 2022).

### 4.3. Variables

**Figure 1:**
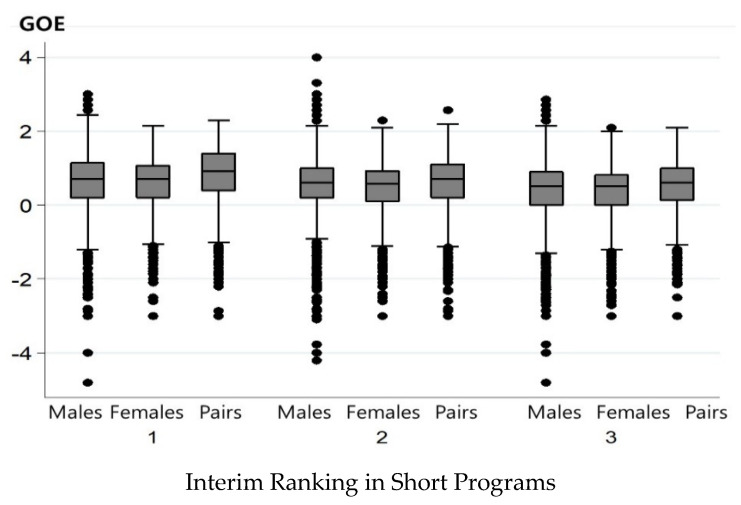
Interim ranking in short programs according to GOE distributions.

Dependent variable. As discussed above, negative GOE scores refer to mistakes in task execution relative to the standard. Hence, this study developed a dummy variable, execution mistakes, which took the value of one if an interim leader earned a negative GOE for the focal task and zero if they earned a positive GOE in the free program. The analysis rested on the premise that there was no difference in the abilities of the interim leaders in the likelihood of executing tasks successfully or poorly. To confirm this premise, Figure 1 presents a box plot to illustrate the insignificant gender and pair bias in the distributions of the GOE scores in the sample.

Independent variables. The independent variables in this study were the two types of pressure-inducing circumstances. First, to capture the pressure from home-field competitions, this study developed a dummy variable that coded as one if the focal competition was held in the interim leader’s home country (home field) and zero if this was not the case. Second, to measure the threat of a ranking drop by a competitive trailing skater, this study identified the trailing skater ranked just below the interim leaders as the nearest trailing skater (NT). As the competitiveness of skaters is based on their performance in previous competitions, interim leaders will obviously notice their NT’s competitiveness if their NT was a gold medalist in a recent competition. Hence, it developed a dummy variable which took the value of one for an NT who won a championship by acquiring a gold medal in a previous competition (gold medalist NT).

Control variables. To eliminate the possibility of alternative explanations, this study incorporated competition-, interim leader- and task-level control variables. First, the study controlled for the characteristics of competitions using two variables. Since skaters usually perform identical programs during a season, learning effects may be present in competitions late in the season. Hence, the study controlled for the number of competitions the interim leaders had participated in during the given season (season experiences). The log-transformed number of skaters were included to control for competition size.

Second, to control for the focal interim leaders’ characteristics, four variables were included. These took into account the fact that the pressure of rank drop on an interim leader may differ depending on their rank. Thus, this study factored the interim leaders’ ranking in the short program (interim ranking). The pressure may also depend on how far the interim leaders’ short program scores are from the trailing skaters below their rank. The smaller these score gaps, the greater would be the pressure perceived by the interim leaders. Hence, the study computed the average differences of interim scores between the interim leaders and their trailing skaters (score gap with trailing skaters). In addition, to control for the pressure for ranking up of the second- and third-place skaters, other skaters ranked ahead of the interim leader are defined as leading competitors (LCs) and those ranked most closely to the interim leader as the nearest leading competitor (NLC). The study then calculated the interim leader’s average score gap with the LCs and recorded the gold medalist NLC in the same manner as with the NTs.

Third, this study controlled for the characteristics of the focal tasks with three variables. Since skaters typically perform poorly in the later parts of programs due to fatigue, it recorded the focal task’s order in the sequences of tasks in the free programs. To control for the level of difficulty in executing tasks, the base values assigned by the ISU for each task were incorporated. Finally, as the skater might be inferior or superior in a certain task, the analysis controlled for the average previous GOE scores of each task in the same season (previous task performance).

### 4.4. Analysis Model

For the dichotomous dependent variable, execution mistakes, the study applied a logistic regression model. All of the model estimations used the logit commands in Stata 17.0. The vce (cluster season) option was included to control for season influences.

The data have a cross-sectional structure. An underlying assumption of the arguments was that competitive pressure placed on the interim leaders influenced their subsequent performance in the free programs. However, poor performance may simply result from lack of inherent capacity or personal characteristics [38]. That is, there may be time-invariant effects of unobservable variables that also accounted for variations in the dependent variables. Hence, individual fixed effects were used to control for unobserved heterogeneity across skaters in all models. Competition and year fixed effects were also used to capture any competition- and year-specific characteristics. Accordingly, the model estimated:

Execution mistakes_itf_ = β1Home field_if_ + β2Gold medalist NT_if_ + X_itf_β + e_itf_,

where Execution mistakes_itf_ is the probability of execution mistakes of the focal task t of focal interim leader i in the focal free program f in a given season, β1 and β2 are scalars and β is a vector of parameters to be estimated, X_itf_ is a vector to control for competition, task and interim leader characteristics, and e_itf_ comprises the unobserved determinants of execution mistakes.

## 5. Results

Table 1 displays the means, standard deviations and correlations of the variables used in the analysis. The correlations between variables were not high. The variance inflation factor (VIF) test for multicollinearity indicated that the largest value was 1.78 across models. This indicated that the models were unaffected by harmful multicollinearity.

The results in Models 1 to 3 in Table 2 show interim leaders ranked in second or third place who may feel pressurized for both ranking increases as well as ranking drops and Models 4 to 6 includes all interim leaders including first rank holders. In each model, the left column shows the results including the control variables only while the right column includes the independent and control variables.

Hypotheses 1 and 2 proposed that female interim leaders in segregated-gender competitions are more likely to make mistakes in task execution than males in pressure-inducing circumstances. The results support Hypothesis 2. A gold medalist NT had a significant influence in preventing female interim leaders from successfully executing their tasks (β = 0.163, *p* < 0.001 in Model 2 and β = 0.107, *p* < 0.05 in Model 5). Contrary to this, the NT affected male interim leaders less significantly, as shown in Models 1 and 4.

However, task performance in home fields as predicted by Hypothesis 1 showed mixed results. In the results shown in Models 1 and 2 in Table 2 (which excludes the first rank), female interim leaders in their home fields made more mistakes (β = 0.113, *p* < 0.05 in Model 2), while male interim leaders, who were exposed to the same environment, made fewer mistakes (β = −0.081, *p* < 0.05 in Model 1). The home-field disadvantage/advantage disappeared when the sample included the first rank, as shown in Models 4 and 5. Therefore, these results provide partial support for Hypothesis 1.

Hypotheses 3 and 4 proposed that the disadvantages experienced by female interim leaders will be less significant in mixed-gender pair competitions. In Models 3 and 6, the coefficients for home field and gold medalist NT were statistically insignificant. Hence, the underperformance induced by competitions held in home fields and the influence of a gold medalist NT did not exist in mixed-gender pair competitions. These results support Hypotheses 3 and 4.

Interestingly, in addition to the above results, the results show that male interim leaders make more mistakes when they are trailing an LC with a large score gap (*p* < 0.05 in Model 1). This contrasts with the result that they are not significantly affected by score gaps with their trailing skaters. The results show that male interim leaders are more sensitive to upward than downward mobility and tend to choke when they perceive the low possibility of upward mobility such as in the case of having large score gaps with the LCs.

To gain additional insights, this study ran the same regressions using the sample of skaters ranked below the three top-most performers in the short programs. If these skaters are less pressured to secure their temporary positions, they will react insignificantly to home-field competitions and to threats from the gold medalist NT across single and pair competitions. The results in Table 3 show that regardless of gender and competition type, lowly-ranked skaters are not significantly influenced by pressure-inducing circumstances. However, the male skaters were still significantly affected by large score gaps with their LCs (*p* < 0.05 in Model 1). Nonetheless, all of these tendencies are insignificant in the mixed-gender pair competitions.

## 6. Discussion

The approach and findings of this study depart from existing relevant studies in at least two ways. First, the majority of previous studies on gender inequality in sports has focused on how gender inequality can exist across diverse contexts and what accelerates such tendencies. For instance, the literature has found that female professional athletes and coaching staff have limited career opportunities or economic incentives compared to males [39,40]. In public, females are also less frequently encouraged to participate in sport events than males [41]. Cultures or stereotypes have reinforced these inequalities [42] and mass media has fed this tendency by emphasizing the femininity of athletes or covering sportspeople unequally [34,43]. This study attempts to shift our focus from these phenomena to solutions that address this inequality by testing how mixed-gender competitions mitigate a gender-specific disadvantage in sports—the heightened competitive pressure on female sportspersons. This approach enriches the literature by facilitating a more balanced discussion of the phenomena, antecedents and solutions to gender inequality in sports.

Second, mixed-gender competitions are a recent transformation in sports [44] and some previous studies had emphasized their importance to achieve gender equality in sports. However, these studies have only emphasized only the participation of female athletes and their performance effects have not been investigated. To address this limitation, some scholars attempted to elucidate such performance effects. For instance, Lee and Cunningham used meta-analysis to find positive effects of group diversity of sports teams on their outcomes, even though the effects were small [45]. Scharfenkamp et al. found that gender-mixed teams tend to outperform all-female teams [46]. The present study supports these existing findings by highlighting the positive impacts of mixed-gender competitions. However, it also extends the ongoing discussions by taking a different approach: alleviation of gender-specific disadvantages such as choking under pressure through gender-mixed competitions.

Furthermore, the results of this study reconcile two critical conflicting arguments in the previous studies. First, the impact of gender on performance under competitive pressure is an important but controversial issue in gender studies. Some scholars have found that females are more sensitive to competitive pressure than males. However, others have shown the opposite or have found complicated/joint influences [6,47]. This study reconciles these conflicting results in the existing literature by suggesting the boundary conditions of female disadvantage under competitive pressure. Specifically, Table 2 and Table 3 show that even in the same tournaments, only highly-ranked female skaters are affected by pressure-inducing circumstances, whereas lowly-ranked skaters are not significantly affected. In addition, disadvantages faced by highly-ranked female skaters disappear in mixed-gender competitions.

Second, this study also challenges the conflicting arguments in previous studies regarding the influence of supportive audiences. Some scholars have found that supportive audiences increase pressure upon performers and thereby negatively impact their performance in diverse contexts such as soccer, golf and laboratory experiments [9,28]. Other scholars, however, have found that supportive audiences cause choking under pressure only in limited contexts or not at all [29,48,49]. Given these caveats, existing research has largely agreed on the contingent effects of supportive audiences but called for further investigation of the contingencies [50]. This study contributes to these discussions by providing evidence for the gender-dependent effects of supportive audiences, indicating that supportive audiences might actually be beneficial for male but not for female interim leaders.

Despite these implications, this study has several limitations, all of which suggest directions for future research. First, this study did not directly measure the degree of pressure skaters perceive but instead made inferences based on certain circumstances. The two circumstances examined in this study represent the situations that impose competitive pressure. However, they are still insufficient in comprehending all possible situations inducing competitive pressures. Future research needs to specify alternative variables such as individual traits [51] or financial incentives [52] and also needs to employ other methodologies to increase the robustness of the results.

Second, this study drew on a single empirical context to examine the impact of gender difference and effect of mixed-gender competitions on competitive pressure. Future research must apply these findings to other sports competitions or experimental conditions as well as alternative factors that can affect the mixed-gender effects to enhance the generalizability of the findings and gain further insights.

Finally, this study only focused on a comparison of interim leader performance between segregated- and mixed-gender competitions. Due to data limitations, it was not possible to compare the performance of mixed- and single-gender team competitions. To deepen our understanding of the effects of mixed-gender competitions, it would be beneficial for future studies to investigate how athletic performance differs under competitive pressure between single- and mixed-gender teams.

## 7. Conclusions

This study conducted an analysis to test the hypothesis that male, female and mixed-gender interim leaders react differently under competitive pressure in dynamic tournaments. Using figure skating as a real-life professional competition, the results confirmed the following with regard to segregated- and mixed-gender competitions: (1) Female interim leaders perform poorly when a highly-qualified trailing skater, specifically a recent gold medalist, tails them most closely. (2) Female interim leaders also underperform when they compete at their home field, but this home disadvantage does not apply to first rank holders. (3) Male interim leaders are not influenced by highly-qualified trailing skaters, and perform better in home-field competitions. (4) Instead, male interim leaders underperform when they are tailing competitors with large score gaps. (5) In mixed-gender competitions, all of these gender-specific influences disappear.

Together, the results proved that gender-specific disadvantages exist in terms of competitive pressure, but they might be mitigated by mixed-gender competitions. Additionally, through the analysis results, this study provides new contingencies to explain existing conflicting arguments surrounding the different gender sensitivity under competitive pressure and the influence of supportive audiences. In this regard, despite the aforementioned limitations, the approach and findings of this study help extend the literature and provide timely advice for the promotion of mixed-gender competitions in practice. The author hopes that these contributions will help increase the awareness in terms of achieving sustainable development in the sports management field.

## Figures and Tables

**Table 1:** 1. Descriptive statistics for the male interim leaders; 2. Descriptive statistics for the female interim leaders; 3. Descriptive statistics for the interim leaders in pair competitions.

	Variable	Mean	SD	1	2	3	4	5	6	7	8	9	10	11
1. Descriptive statistics for the male interim leaders														
1	Execution mistakes	0.19	0.39											
2	Season experiences	2.35	1.22	−0.03										
3	Number of skaters	14.44	6.70	−0.02	0.59 *									
4	Interim ranking	2.50	0.50	0.06 *	0.03 *	0.05 *								
5	Score gap with LCs	5.40	3.88	0.04	−0.05 *	−0.03	0.10 *							
6	Score gap with trailing skaters	14.05	5.49	−0.04 *	0.39 *	0.66 *	−0.16 *	−0.19 *						
7	Gold medalist NLC	0.22	0.41	−0.03	0.25 *	−0.02	−0.13 *	0.05 *	−0.03					
8	Sequence	1.72	0.75	−0.23 *	0.02	0.02	0.00	0.00	0.02	0.00				
9	Base values	5.66	3.22	0.27	0.06	0.00	0.00	0.02	0.05	0.02	−0.46 *			
10	Previous task performance	0.26	0.82	−0.36 *	0.11 *	0.06 *	−0.05 *	−0.01	0.09 *	0.08 *	0.26 *	−0.19		
11	Home field	0.20	0.40	−0.03	−0.19 *	−0.20 *	−0.01	0.13 *	−0.15 *	0.00	0.00	0.01	−0.01	
12	Gold medalist NT	0.14	0.35	−0.01	0.31 *	0.07 *	−0.15 *	0.04	0.05 *	0.11 *	0.02	0.00	0.06 *	−0.01
2. Descriptive statistics for the female interim leaders														
1	Execution mistake	0.20	0.40											
2	Season experiences	2.47	1.20	−0.07 *										
3	Number of skaters	14.64	6.64	−0.09 *	0.56 *									
4	Interim ranking	2.49	0.50	0.02	−0.05 *	0.00								
5	Score gap with LCs	3.31	2.84	0.02	−0.18 *	−0.09 *	0.18 *							
6	Score gap with trailing skaters	11.12	4.85	−0.06 *	0.28 *	0.69 *	−0.17 *	−0.18 *						
7	Gold medalist NLC	0.27	0.44	−0.02	0.34 *	0.01	−0.15 *	0.00	0.03					
8	Sequence	1.67	0.73	−0.17 *	0.01	0.01	0.01	0.00	0.01	0.01				
9	Base values	4.49	2.13	0.14 *	0.06 *	0.02	−0.01	0.01	0.02	0.02	−0.40 *			
10	Previous task performance	0.27	0.66	−0.41 *	0.13 *	0.08 *	−0.02	−0.04 *	0.10 *	0.04	0.20 *	−0.24 *		
11	Home field	0.18	0.38	0.00	−0.04	0.03	0.02	0.18 *	0.05 *	0.05 *	0.01	−0.01	0.01	
12	Gold medalist NT	0.12	0.32	0.05 *	0.14 *	−0.19 *	−0.06 *	−0.11 *	−0.17 *	0.12 *	0.00	0.00	0.02 *	−0.12
3. Descriptive statistics for the interim leaders in pair competitions														
1	Execution mistake	0.16	0.36											
2	Season experiences	2.58	1.33	−0.05 *										
3	Number of skaters	9.98	4.10	−0.05 *	0.65 *									
4	Interim ranking	2.48	0.50	0.00	0.01	0.02								
5	Score gap with LCs	4.79	3.50	0.04	−0.22 *	−0.17 *	0.24 *							
6	Score gap with trailing skaters	11.36	4.61	−0.04	0.31 *	0.59 *	−0.20 *	−0.23 *						
7	Gold medalist NLC	0.36	0.48	−0.02	0.19 *	0.04 *	−0.19 *	−0.06 *	0.02					
8	Sequence	1.68	0.72	−0.23 *	0.00	0.00	0.02	0.02	0.00	−0.01				
9	Base values	4.73	1.60	−0.06 *	0.02	0.02	−0.02	−0.05 *	−0.01	0.00	−0.11 *			
10	Previous task performance	0.35	0.70	−0.46 *	0.07 *	0.03	−0.05 *	−0.09 *	0.00	0.05 *	0.18 *	0.13 *		
11	Home field	0.21	0.41	0.00	−0.17 *	−0.11 *	−0.02	−0.01	−0.11 *	−0.01	0.00	0.00	−0.06 *	
12	Gold medalist NT	0.08	0.28	−0.04	0.21 *	0.07 *	−0.10 *	−0.12 *	−0.12 *	0.21 *	−0.01	0.04	0.08 *	−0.06 *

* *p* < 0.05.

**Table 2:** Analysis results for execution mistakes of the interim leaders excluding the first rank.

**Variables**	**Model 1 (Males)**				**Model 2 (Females)**				**Model 3 (Pairs)**			
Season experiences	−0.152		−0.171		0.111		0.107		−0.133		−0.130	
	(0.15)		(0.15)		(0.12)		(0.12)		(0.21)		(0.21)	
Number of skaters	0.408		0.336		−0.41		−0.448		0.227		0.216	
	(0.47)		(0.47)		(0.57)		(0.50)		(0.33)		(0.32)	
Interim ranking	1.529		1.747	†	1.016		1.019		0.476		0.428	
	(1.06)		(1.01)		(0.79)		(0.77)		(0.71)		(0.72)	
Score gap with LCs	0.334	**	0.341	**	−0.105		−0.105		0.199		0.184	
	(0.12)		(0.12)		(0.12)		(0.13)		(0.14)		(0.15)	
Score gap with trailing skaters	−0.101		−0.106		0.077		0.042		0.129		0.123	
	(0.11)		(0.11)		(0.19)		(0.16)		(0.16)		(0.16)	
Gold medalist NLC	0.005		0.021		−0.020		−0.023		−0.040		−0.037	
	(0.06)		(0.06)		(0.04)		(0.05)		(0.07)		(0.07)	
Sequence	−0.111		−0.113		−0.213	**	−0.218	**	−0.427	***	−0.427	***
	(0.08)		(0.08)		(0.07)		(0.07)		(0.08)		(0.08)	
Base values	0.542	***	0.544	***	0.032		0.032		−0.161	†	−0.162	†
	(0.07)		(0.07)		(0.13)		(0.13)		(0.09)		(0.09)	
Previous task performance	−0.701	***	−0.702	***	−1.266	***	−1.277	***	−1.401		−1.404	***
	(0.07)		(0.07)		(0.13)		(0.12)		(0.11)		(0.11)	
Home field			−0.081	*			0.113	*			−0.053	
			(0.04)				(0.06)				(0.09)	
Gold medalist NT			0.064				0.163	***			−0.009	
			(0.05)				(0.04)				(0.06)	
Constant	0.584		0.808		−0.712		−0.972		−0.802		−0.923	
	(1.26)		(1.13)		(0.88)		(0.88)		(0.90)		(1.00)	
Skater fixed effects	Yes		Yes		Yes		Yes		Yes		Yes	
Year fixed effects	Yes		Yes		Yes		Yes		Yes		Yes	
Competition fixed effects	Yes		Yes		Yes		Yes		Yes		Yes	
Log pseudo-likelihood	−886.726		−885.262		−833.476		−827.797		−670.44		−670.44	
Pseudo-R2	0.221		0.222		0.214		0.220		0.294		0.294	
N	2365		2365		2129		2129		2194		2194	
**Variables**	**Model 4 (Males)**				**Model 5 (Females)**				**Model 6 (Pairs)**			
Season experiences	−0.073		−0.074		−0.003		0.005		0.008		0.007	
	(0.12)		(0.12)		(0.10)		(0.10)		(0.15)		(0.15)	
Number of skaters	0.257		0.261		−0.371		−0.334		0.233		0.223	
	(0.32)		(0.32)		(0.38)		(0.37)		(0.19)		(0.18)	
Interim ranking	0.564		0.598		0.08		0.266		0.419		0.37	
	(0.51)		(0.52)		(0.48)		(0.46)		(0.34)		(0.33)	
Score gap with trailing skaters	−0.092		−0.102		−0.107		−0.116		−0.102		−0.097	
	(0.08)		(0.07)		(0.13)		(0.12)		(0.15)		(0.15)	
Sequence	−0.061		−0.061		−0.264	***	−0.265	***	−0.386	***	−0.386	***
	(0.06)		(0.06)		(0.04)		(0.04)		(0.07)		(0.07)	
Base values	0.469	***	0.471	***	0.021		0.019		−0.273	***	−0.273	***
	(0.05)		(0.06)		(0.12)		(0.12)		(0.06)		(0.06)	
Previous task performance	−0.689	***	−0.688	***	−1.270	***	−1.276	***	−1.336	***	−1.336	***
	(0.06)		(0.06)		(0.08)		(0.08)		(0.08)		(0.08)	
Home field			−0.002				−0.003				−0.039	
			(0.06)				(0.05)				(0.07)	
Gold medalist NT			0.041				0.107	*			−0.028	
			(0.04)				(0.04)				(0.03)	
Constant	−1.604	*	−1.603	*	−1.483	*	−1.302	*	−1.014		−1.101	
	(0.71)		(0.71)		(0.64)		(0.64)		(0.85)		(0.84)	
Skater fixed effects	Yes		Yes		Yes		Yes		Yes		Yes	
Year fixed effects	Yes		Yes		Yes		Yes		Yes		Yes	
Competition fixed effects	Yes		Yes		Yes		Yes		Yes		Yes	
Log pseudo-likelihood	−1371.70		−1370.94		−1221.63		−1217.76		−1006.82		−1006.54	
Pseudo-R2	0.187		0.187		0.226		0.228		0.278		0.279	
N	3591		3591		3278		3278		3418		3418	

† *p* < 0.1, * *p* < 0.05, ** *p* < 0.01, *** *p* < 0.001. Note: Standard errors in parentheses. All variables standardized for comparison.

**Table 3:** Analysis results for execution mistake of lowly-ranked skaters.

Variables	Model 1 (Males)		Model 2 (Females)		Model 3 (Pairs)	
Season experiences	0.107	**	0.116	*	−0.110	†
	(0.04)		(0.05)		(0.06)	
Number of skaters	0.101		−0.030		−0.169	
	(0.20)		(0.21)		(0.14)	
Interim ranking	0.078		0.265	***	0.099	
	(0.06)		(0.06)		(0.11)	
Score gap with LCs	0.166	*	0.075		0.102	
	(0.07)		(0.06)		(0.09)	
Score gap with trailing skaters	−0.104		0.058		−0.014	
	(0.09)		(0.07)		(0.07)	
Gold medalist NLC	−0.024		0.004		0.029	
	(0.04)		(0.03)		(0.09)	
Sequence	−0.023		−0.274	***	−0.246	***
	(0.04)		(0.02)		(0.06)	
Base values	0.399	***	0.089		−0.215	***
	(0.03)		(0.08)		(0.05)	
Previous task performance	−0.853	***	−1.301	***	−1.319	***
	(0.05)		(0.06)		(0.07)	
Home field	−0.049		−0.053		0.010	
	(0.03)		(0.04)		(0.05)	
Gold medalist NT	0.076		0.008		−0.074	
	(0.05)		(0.06)		(0.06)	
Constant	−0.765	*	0.306		−0.676	*
	(0.39)		(0.37)		(0.29)	
Skater fixed effects	Yes		Yes		Yes	
Year fixed effects	Yes		Yes		Yes	
Competition fixed effects	Yes		Yes		Yes	
Log pseudo-likelihood	−4807.85		−4251.68		−2059.03	
Pseudo-R2	0.196		0.230		0.263	
N	10,477		9062		4998	

† *p* < 0.1, * *p* < 0.05, ** *p* < 0.01, *** *p* < 0.001. Note: Standard errors in parentheses. All variables standardized for comparisons.

## Data Availability

Not available.

## Appendix A

In the context of figure skating competitions, each competition consists of two subprograms, the short and free programs, held over two days. On the first day, skaters execute 7 skating techniques in the short program and on the second day, they execute 14 techniques in the free program. Throughout the programs, skaters must execute four or five generic types of techniques in both the single and pair competitions. These include spins, step sequences, spirals, jumps and lifts (pair competition only). Each type of technique has multiple subcategories. For example, jumps have six subcategories that are characterized by takeoff, edging, rotating and landing, namely, toe loop (T), Salchow (S), loop (L), flip (F), lutz (Lz) and axel (A). These techniques can be executed independently (e.g., a triple loop, denoted by 3Lo) or jointly (a triple lutz and a double loop, denoted by 3Lz + 2Lo) and each requires different types of skills and has varying levels of difficulty. Each of these independent or joint techniques constitutes a task in this context.

Skaters develop their own short and free programs with multiple tasks and execute the programs in each of the competitions during a season. In each competition, a final ranking is determined based on the total score that includes the total element scores and the program components scores for the short and free programs. The element score is the sum of the points for each task performed, whereas holistic aspects of a program such as choreography or interpretation evaluate the program components score. To examine the task performance of skaters, this study focused on the element scores as they directly and objectively measure how well skaters perform the tasks in the specified ways.

This context is highly suitable for developing an argument about gender differences and effects of mixed-gender under competitive pressure. First, figure skating competition is a representative dynamic tournament. At the end of the short program, all participants know their interim rankings. This imposes greater pressure on skaters to perform in the free programs as the final rankings will change depending on how much better (or worse) they perform on the second day. Hence, by looking at the temporary ranking after the short programs and subsequent performance of skaters in the free programs, it is possible to examine how the pressure to secure final high rankings affect subsequent task performance.

Second, this context is a representative mixed-gender sport that includes segregated events for male and female single competitions as well as pair competitions. Despite the gender diversity of participants, each of these competitions has the same structure and common evaluation systems that depend on similar basic techniques. These commonalities are effective while comparing skater performance across single/pair competitions as well as for the different genders. Finally, unlike most other sports competitions, the skaters in this context develop their programs and practice them before actual execution. This suggests that skater performance is likely to reflect actual mental conditions and psychological processes, such as competitive pressure at the point of execution, as this study assumes.
